# Median suboccipital craniotomy and telovelar approach for posterior pontine cavernous malformations

**DOI:** 10.3171/2019.7.FocusVid.19134

**Published:** 2019-07-01

**Authors:** Daniel D. Cavalcanti, Paulo Niemeyer Filho

**Affiliations:** 1 Department of Neurosurgery, Barrow Neurological Institute, St. Joseph’s Hospital and Medical Center, Phoenix, Arizona; and; 2 Department of Neurosurgery, Paulo Niemeyer State Brain Institute, Rio de Janeiro, Rio de Janeiro, Brazil

**Keywords:** brainstem cavernous malformation, pons, median suboccipital craniotomy, telovelar approach, safe entry zone, microsurgery, video

## Abstract

The pons is the preferred location for cavernous malformations in the brainstem. When these lesions do not surface, it is critical to select the optimal safe entry zone to reduce morbidity.^1–3^ In this video, we demonstrate in a stepwise manner the medial suboccipital craniotomy and the telovelar approach performed in a lateral decubitus position. They were used to successfully resect a pontine cavernous malformation in a centroposterior location in a 19-year-old patient with diplopia, right-sided numbness, and imbalance. The paramedian supracollicular safe entry zone was used once the lesion did not reach the ependymal surface.^2,3^ Late magnetic resonance imaging demonstrated total resection and the patient was neurologically intact after 3 months of follow-up. The approach is also demonstrated in a cadaveric dissection to better illustrate all steps.

The video can be found here: https://youtu.be/ChArkxA8kig.

## Transcript

Median suboccipital craniotomy and telovelar approach for posterior pontine cavernous malformations.

This is the history of a 19-year-old male with acute episode of headaches 3 weeks prior admission, double vision, progressive imbalance, and numbness in right face and arm.

In the physical exam, it was found reduced sensation to light touch and pinprick in the right hemiface and arm, as well as right cranial nerve VI palsy.

Located in the central pons, there is a round lesion hypointense to isointense centrally (0:45). Posteriorly, it is possible to observe the classic popcorn aspect, with the lesion markedly heterogeneous in signal with regions of high signal in both T1 and T2 images.

There is a peripheral signal dropout better seen in gradient echo sequence, consistent with hemosiderin.

The surgical technique will be first demonstrated in a cadaveric model (1:09). In the lateral decubitus position, we use a straight midline skin incision from the inion down to the C2 spinous process. The dissection is extended through the deep layers to the level of the suboccipital bone and the posterior tubercle of C1, along the midline avascular fascial planes. The muscles are elevated from the underlying occipital bone and posterior arch of C1 in a subperiosteal fashion. Two lateral burr holes are placed laterally intersecting the inferior edge of the transverse sinus to complete the suboccipital craniotomy. The craniotome with the footplate should carefully cross the internal occipital crest. The bone flap is carefully elevated, and a laminotomy of the posterior arch of C1 is complete to increase the vertical angles of approach to the rhomboid fossa.

The dura is then opened in a Y-shaped fashion to preserve the occipital sinus. The cisterna magna is opened to obtain relaxation of the cerebellum, to expose bilateral tonsils and the cerebellomedullary fissure.

The tonsils are gently retracted (02:12), the tela choroidea is divided, and the inferior medullary velum is exposed and divided to fully expose the floor of the fourth ventricle. The floor of the fourth ventricle is a very eloquent area and whenever a cavernous malformation does not arise on the ventricular floor of the fourth ventricle, it is critical to understand the anatomy of the safe entry zones. Four main safe entry zones are located around the facial colliculi.

We believe that the lateral decubitus (2:38) can provide the same exposure of the floor of the fourth ventricle as the prone and the semisitting position without all the respiratory drawbacks of those positions. The upper torso of the patient is elevated up to 30° off the floor and the skin incision is kept almost parallel to the floor. All the pressure points are padded and the patient is safely secured to the table. The dependent shoulder and arm are rested in a padded armboard while the nondependent one is slightly retracted down to improve surgical freedom. The head is then fixed with a three-pin head holder clamp.

After prepping and draping the patient in sterile fashion, the skin incision was done in a vertical line from the inion to the C2 spinous process. The skin and the muscular layers are retracted with two cerebellar retractors (3:25), and with a one-burr-hole craniotomy done in this patient with the burr hole sitting just below the projection of the torcula on the surface. The bone flap is elevated and the dura is opened in a Y-shaped fashion.

Opening the arachnoid membrane overlying the cisterna magna (3:42) will relax the cerebellum, expose both tonsils as well as the cerebellomedullary fissure. Gentle retraction of the nondependent tonsil (3:50) as well as the tonsilomedullary segment of the PICA will immediately expose the inferior half of the floor of the fourth ventricle. A wide dissection of bilateral cerebellomedullary fissures, releasing the arachnoid membranes, is key to release both tonsils and expose the tela choroidea. Opening the tela choroidea (4:11), while releasing the distal segments of PICA, will enhance cranial exposure of the floor of the fourth ventricle, while reducing manipulation of that vessel. At this point, image guidance is indispensable for locating a lesion that does not come up on the ependymal surface. At this point, we combine the knowledge of the anatomy of the safe entry zones of the brainstem, the intraoperative monitoring of the seventh nerve, and the image guidance.

The neurotomy (4:37) consisted in a very small vertical sharp incision over the suprafacial triangle or also called paramedian supracollicular area. The dissection (4:47) was carried out in a vertical way parallel to the ascending and descending tracts, minimally stretching the fibers. After evacuating the fluid hematoma and decompressing the brainstem, irrigation can help improving the visualization of the deep cavity. Removal of the cavernous malformation and blood products in different stages of evolution is done in a piecemeal fashion.

Absolutely slight traction is done to pull out small fragments of the lesion. Care should be taken to keep the dissection within the limits of the lesion. Frequent use of image guidance and intraoperative monitoring help reducing inadvertent injuries to the surrounding tracts. Different 1-mm cup forceps are used to grasp and pull fragments of the cavernous malformation (5:38). It is key to have optimal illumination in most of the corners of the operative bed in order to achieve gross-total resection and prevent transgression of the surrounding hemosiderin-laden parenchyma as well as damage to developmental venous anomalies.

Some sinusoids with active venous flow can be coagulated with bipolar forceps using low-power coagulation.

The microsuction in the nondominant hand is used most of times, during the resection, as a countertraction tool. It is important to constantly recheck the position of the facial nerve fibers (6:31). Changing the orientation of the dissection allowed us to expose a large fragment of the cavernous malformation. This large compartment of the cavernous malformation has not only patent sinusoids but also hematomas in different stages of resolution. The significant part is finally resected (7:02). Once again, piecemeal resection is strongly advised to large, deep-seated, cavernous malformations.

Dividing large dissected fragments (7:19), still attached to surrounding parenchyma, significantly reduces damage to adjacent nuclei and tracts. Another fragment of cavernous malformation is seen being removed (7:35). The microsurgical cavity is inspected for residuals (7:45). A hemostatic agent is infused into the cavity and left in place for 2 minutes. Most of it is aspirated and bleeding points are coagulated with bipolar forceps in minimal power.

Watertight closure of the dura is carried out with 4-0 Prolene sutures (7:57).

The patient had slight facial weakness in bilateral lower quadrants in the immediate postoperative period. The upper face was preserved. At the 3-month follow-up, the patient was neurologically intact and the MRI demonstrated complete resection without any residuals (8:11).

The 1-year follow-up visit confirmed the good outcome.
